# Effects of Internet-Based Support Program on Parenting Outcomes for Primiparous Women: A Pilot Study

**DOI:** 10.3390/ijerph18094402

**Published:** 2021-04-21

**Authors:** Lingling Huang, Qu Shen, Qiyu Fang, Xujuan Zheng

**Affiliations:** 1 Health Science Centre, Shenzhen University, Shenzhen 518060, China; huanglingling@szu.edu.cn (L.H.); fangqiyu320@szu.edu.cn (Q.F.); 2 Department of Nursing, School of Medicine, Xiamen University, Xiamen 361102, China; shenqumail@163.com

**Keywords:** pilot study, internet-based intervention, primiparous women, maternal self-efficacy, postpartum depression, social support

## Abstract

(1) *Background:* Some primiparous women are usually confronted with many parenting problems after childbirth, which can negatively influence the wellbeing of some mothers and infants. Evidence identified that internet interventions can include more tailored information, reach a larger research group, and supply more anonymity than face-to-face traditional interventions. Therefore, the internet-based support program (ISP) was designed to improve the parenting outcomes for Chinese first-time mothers. (2) *Methods*: A multicenter, single-blinded, pilot randomized controlled trial was conducted. From May to October 2020, a total of 44 participants were recruited in the obstetrical wards of two tertiary hospitals in China. Eighteen women in the control group received routine postnatal care; while eighteen women in the intervention group accessed to the ISP and routine postnatal care. The duration of intervention was not less than three months. Intervention outcomes were assessed through questionnaires before randomization (T0), immediately after intervention (T1), and three months after intervention (T2). The Self-efficacy in Infant Care Scale (SICS), Edinburgh Postnatal Depression Scale (EPDS), and Postpartum Social Support Scale (PSSS) were included to measure MSE, postpartum depression (PPD), and social support, respectively. (3) *Results:* No significant difference between the two groups were found in terms of the baseline social-demographic characteristics; and the scores of SICS, EPDS and PSSS at T0 (*p* > 0.05). Repeated measures multivariate analysis of covariance found that women in the intervention group had a higher MSE score at T1 (6.63, *p* = 0.007), and T2 (5.75, *p* = 0.020); a lower EPDS score at T1 (3.11, *p* = 0.003), and T2 (2.50, *p* = 0.005); and a higher PSSS score at T1 (4.30, *p* = 0.001); and no significant difference at T2 (0.35, *p* = 0.743), compared with women in the control group. (4) *Conclusion*: The effect of ISP was evaluated to significantly increase primiparous women’s MSE, social support, and to alleviate their PPD symptoms. However, the small sample in pilot study restricted the research results. Therefore, the ISP should be further investigated with a larger, diverse sample to confirm whether it should be adopted as routine postnatal care to support primiparous women on parenting outcomes and mental wellbeing in the early stage of motherhood.

## 1. Introduction

Due to the lack of parenting experience, some primiparous women frequently suffer from various parenting problems, such as unsuccessful parenting tasks and negative mother–infant interaction in the early stage of motherhood, which can negatively affect the physical and mental wellbeing of some mothers and infants [1,2,3]. As a significant indicator of parenting outcomes [3], maternal self-efficacy (MSE) refers to the belief that women hold of their capability about the performance of various parenting tasks [4]. Studies found that Chinese primiparous women had a lower level of MSE, compared with the samples in the USA [5], UK [6], Canada [7], and Finland [8,9].

Sound evidence identifies that some factors impact on MSE; and the main variables influencing MSE are postpartum depression (PPD) and social support [2,10,11]. Research showed that in comparison with Western women, Chinese new mothers were more prone to have PPD due to the high expectations of motherhood in Chinese culture and dealing with the sensitive relationship with mothers-in-law [12,13]. In terms of social support, Chinese primiparous women were reported to receive insufficient social support after delivery, and especially lacked adequate informational and appraisal support from health professionals on women’s parenting tasks [13].

Some interventions were undertaken in order to improve primiparous women’s parenting outcomes and mental health. For instance, the effects of mindfulness intervention on MSE, maternal wellbeing and mental distress were evaluated by a RCT (randomized controlled trial) for Spanish mothers [14]. The intervention was delivered during an eight-week meditation course, with a two-hour session once a week. Research found that primiparous women in the intervention group had a significantly higher MSE score, and experienced significantly less anxiety and stress, than women in the control group. The other RCT conducted in Mainland China was to explore the outcomes of an interpersonal psychotherapy-oriented education program including two 90-min face-to-face educational group meetings and a follow-up telephone during two weeks postpartum. In comparison of the control group, women in the study group were reported to have a significantly higher score of MSE, social support, and a lower score of PPD at 12 weeks postpartum [12,15]. 

These traditional face-to-face interventions were identified to have an effect in the improvement of parenting outcomes [10]; however, the large number of Chinese primipara, the inadequate funding for maternal health care and the deficiency of Chinese health professionals pose great challenges to the accessibility of these traditional approaches [16]. Therefore, the innovative, effective and feasible intervention methods should be considered. Evidence identified that internet interventions can include more tailored information, approach larger research population, and supply more anonymity than face-to-face traditional interventions [17,18,19,20,21]. In 2020, China News reported that the internet by a device of mobile or computer was available to 61% of the Chinese population [22]. To our knowledge, there has been no RCTS on parenting outcomes in Chinese primiparous women via the Internet [10]. Therefore, the internet-based support program (ISP) was designed to improve the Chinese primiparous women’s parenting ability, mental wellbeing, and social support.

## 2. Materials and Methods

### 2.1. Study Design

The study protocol was published in JAN [10]. In this study, a multicenter, single-blinded, pilot RCT (randomized controlled trial) was used to assess the effects of ISP for Chinese first-time mothers during the early stage of motherhood, in regarding of the improvements of MSE, and social support; and the alleviation of PPD symptoms. 

Ethical approval (2020011) was obtained from Shenzhen University. The trial was registered with the Chinese Clinical Trial Registry (ChiCTR2000033154). The researchers obtained all participants’ consent form prior to data collection; and informed them of freedom to withdraw whenever they want. All collected data were kept anonymously and confidentially. 

### 2.2. Participants

Eligible women met the criteria of 18 years old or above; being first-time mothers with healthy babies; having ability to response the questionnaires; and being available to the internet by mobile phone or computer. Women were excluded if they or their infants had serious diseases. From May to October 2020, a total of 98 women were approached, and 30 women were excluded as they did not meet the inclusion criteria, and 24 of them were declined. Finally, 44 participants were recruited when primiparous women admitted in maternity wards of two public tertiary hospitals in China; and were randomly assigned into the control group and the intervention group with allocation ratio as 1:1. Blinding was not possible for the researchers during all the research process; however, the group allocation was masked during the recruitment until the baseline measures are completed. Trial participants, outcome assessors, and data analysts were blinded to the participants’ group allocation. 

### 2.3. Intervention

**Figure 1:**
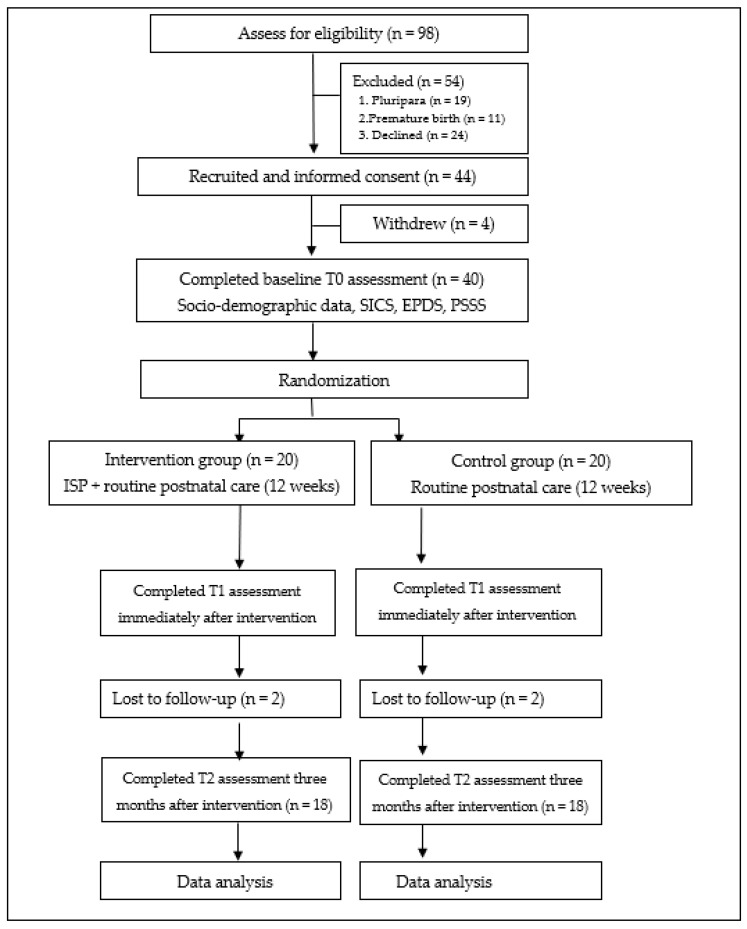
CONSORT (Consolidated Standard of Reporting Trials) flowchart of the Internet-based Support Program (ISP). SICS: the Self-efficacy in Infant Care Scale; EPDS: the Edinburgh Postnatal Depression Scale; PSSS: the Postpartum Social Support Scale.

Women in the control group received routine postnatal care; while women in the intervention group accessed to the ISP and routine postnatal care. Routine postnatal care consists of supports from the obstetricians and obstetric nurses during the 3–5 days hospitalization; and home visits from the community doctors on the 3rd, 7th, 14th, and 28th days postpartum [1]. The duration of intervention was not less than three months. Intervention outcomes were investigated via questionnaires before randomization (T0), immediately after the intervention (T1), and three months after the intervention (T2). The CONSORT [23] flowchart was illustrated in Figure 1. The ISP was developed according to the self-efficacy theory and the social exchange theory, which has five components including learning forum, communication forum, ask-the-expert forum, baby home forum, and reminder forum [10]. The detailed contents of the ISP were shown in Table 1. Participants in the intervention group were taught by the researchers to log in and use each components of the ISP. The duration and frequency of the logins were monitored to assess the adherence of participants. Reminder telephones or Wechat were conducted every week for reminding intervention group women to use the ISP, such as frequency logins at least twice one week, and total usage time no less than one hour per week.

### 2.4. Outcomes

#### 2.4.1. Primary Outcomes

The primary outcome was MSE comparing the intervention and the control group at T1 and T2. MSE was assessed by the Self-efficacy in Infant Care Scale (SICS) [24]. It is a 46-item self-reported tool to assess the belief of women in their capability about the performance of various parenting tasks. Each item means one parenting task ranging from 0-100 points; and the higher mean score of SICS indicates the higher level of MSE. The internal consistency of SICS (Cronbach alpha = 0.95) was good for this study.

#### 2.4.2. Secondary Outcomes

The secondary outcomes of this pilot study were PPD and social support. PPD was evaluated via the Edinburgh Postnatal Depression Scale (EPDS) [25]. The instrument has ten items using four-point Likert score; and its total score ranges from 0 to 30. The lower score means the better mental health status women have. The reported Cronbach’s alpha coefficient of Chinese version EPDS was 0.87; and its concurrent validity was 0.79 with the BDI (Beck Depression Inventory) [26]. The internal consistency of the EPDS was 0.85 in the current research. 

Social support was measured through the Chinese version of Postnatal Social Support Scale (PSSS). The 20-item instrument was to evaluate women’s perception of support after delivery. The total score of PSSS ranges between 0 and 60 points; and a higher score the mother has, indicates the more social support she receives. The internal consistency of this tool was 0.89 [27]. In the present study, the Cronbach’s alpha coefficient of PSSS was 0.90.

#### 2.4.3. Other Outcomes

The baseline social-demographic characteristics of participants included the women’s age, marital status, education, occupation, monthly family income, delivery mode, whether attending parenting training before childbirth, infant gender, infant health and infant fussiness by women’s self-report.

### 2.5. Data Collection

The baseline assessment was conducted by the research team, and every participant completed the social-demographic data, SICS, EPDS, and PSSS in the maternity wards. At T1 and T2, the electronically questionnaires of SICS, EPDS, and PSSS were distributed to participants using email or WeChat; and the completed questionnaires were returned to the research team likewise via email or WeChat (Tencent, Shenzhen, China). In order to improve the response rate, participants received a kindly telephone or WeChat reminder before and after one week of T1 and T2 assessment.

### 2.6. Data Analysis

The Statistical Package for Social Sciences (SPSS, IBM Corp, New York, USA) 22.0 was used for the data analysis. Descriptive statistics were used to describe the social-demographic characteristics and clinical variables by mean (M), standard deviation (SD), and frequency, proportions. To detect any significant difference between the intervention group and the control group on the social-demographic characteristics and baseline outcomes, the chi-square (χ^2^) for categorical variables and the independent sample t-test for continuous variables were used. The effect of intervention in terms of SICS, EPDS and PSSS was evaluated via repeated measures multivariate analysis of covariance to explore how outcomes has changed between groups; over time, and the interaction between group and time, adjusted through the corresponding baseline values.

## 3. Results

### 3.1. Baseline Characteristics

In the pilot study, 44 first-time mothers were recruited, and 40 of them completed baseline assessment and were randomly allocated into the two group. Finally, 18 women in the study group and 18 women in the control group completed the follow-up measurement (response rate: 81.8%). 

No significant difference was found between the intervention and the control groups in terms of the baseline social-demographic characteristics; and the scores of SICS, EPDS and PSSS (*p* > 0.05) (Table 2). 

### 3.2. Effectiveness of the ISP

Research found that compared with the control group, women in the intervention group had a higher MSE score at T1 (mean difference = 6.626, *p* = 0.007), and T2 (mean difference = 5.746, *p* = 0.020); a lower EPDS score at T1 (mean difference = 3.111, *p* = 0.003), and T2 (mean difference = 2.500, *p* = 0.005); and a higher social support score at T1 (mean difference = 4.302, *p* = 0.001); and no significant difference at T2 (mean difference = 0.350, *p* = 0.743) (Table 3).

**Figure 2:**
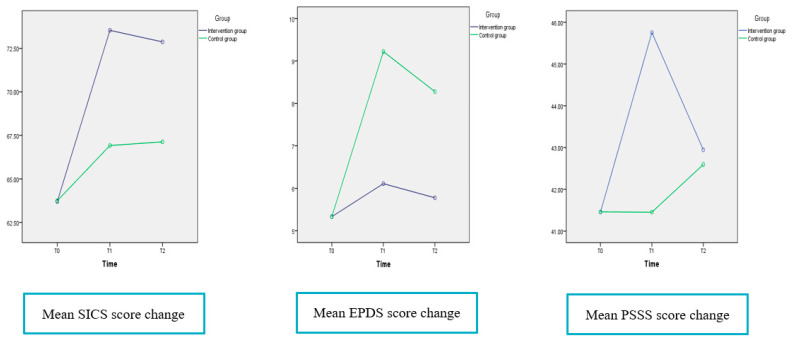
The mean score change in parenting outcomes of MSE, postpartum depression and social support. SICS: the Self-efficacy in Infant Care Scale; EPDS: the Edinburgh Postnatal Depression Scale; PSSS: the Postpartum Social Support Scale.

In both two groups, the MSE score of participants increased from T0 to T1, T0 to T2 (*p* <0.01); and the increase was significantly higher for the intervention group women than for the control group women. There was no significant difference of EPDS score in the intervention group with the passage of time; however, there was the significant increased EPDS score in control group from T0 to T1 (mean difference = 3.889, *p* < 0.001), T0 to T2 (mean difference = 2.944, *p* < 0.001). The social support score in the intervention group had a significant increase from T0 to T1 (mean difference = 4.298, *p* < 0.001), and T0 to T2 (mean difference = 1.488, *p* = 0.001). By comparison, no such increase in social support score was found in control group as time went on. Figure 2 showed a graphical representation of the mean score change in parenting outcomes. 

## 4. Discussion

The objective of this pilot study was to assess the effects of internet-based support program (ISP) to improve the Chinese primiparous women’s parenting ability, mental wellbeing, and social support. Upon completing the ISP, primiparous women in the study group had a higher MSE score at T1 and T2; a lower EPDS score at T1, and T2; and a higher PSSS score at T1, in comparison with women in the control group. 

The pilot study suggested that this ISP had the potential to improve the MSE level for primiparous women; and these beneficial effects could be sustained at three months after intervention, in keeping with previous research using face-to-face intervention methods [12,14,15]. Chinese primiparous women were reported to suffer from many parenting problems of unsuccessful parenting tasks and inefficient mother-infant interactions, significantly undermined the physical and mental wellbeing of women and infants [1,2]. In order to improve the parenting outcomes of new mothers, the intervention of ISP includes the various parenting knowledge and skills that taught via attractively multimedia resources from the learning forum and baby home forum; and the sharing parenting feeling and experience from the communication forum; and the instructional feedback from health professionals in the ask-the-expert forum; and the kindly suggestions from other mothers in the communication forum. These learning materials, sharing parenting experiences and various kinds of supports could effectively improve new mothers’ parenting ability and confidence, supported by Bandura’s theory [28]. The current research showed that in both two groups, the MSE score of participants increased from T0 to T1, T0 to T2, which were consistent with the prior study results that the mean MSE scores of women increased with the passage of time [2,29,30]. However, the increase was significantly higher for the intervention group women than the control group women. The results likewise demonstrated the effect of ISP on the improvement of MSE levels.

The previous studies found that a higher proportion of Chinese primiparous women were prone to have PPD, compared with the women in Western country [1,2]. In this research, the ISP was identified to significantly alleviate PPD for first-time mothers in the intervention group; and the positive outcomes on PPD could be sustained at three months after intervention. As researchers argued that the ISP could improve women’s psychological wellbeing by the promotion of mothers’ capability to successfully fulfill varied parenting tasks; initiatively mediate their mood and positively deal with the relationship with mother-in-law [10]. In our study, there was the significant increased EPDS score in control group from T0 to T1, and the decreased EPDS score from T1 to T2, which was consistent with the previous research [2,30]. As reported, PPD was identified to have a peak incidence at about six to eight weeks postpartum; and its symptoms would remit with the passage to time [31,32]. However, the alleviation of PPD as time went on was too little to have clinical significance [1,2]. Thus, the ISP is strongly recommended to improve primiparous women ‘s mental status after childbirth.

In the present study, the ISP was reported to enhance social support level for primiparous women; however, the effects were not sustained at three months after intervention, inconsistent with the prior findings [12,15]. For example, women in the intervention group had a higher social support score at T1 than women in the control group; but no significant difference at T2. No longer-term effects of the ISP on social support for primiparous women may be because the sample of pilots study was too small to identify the outcomes on social support. Moreover, the possible explanation was that women could access the ISP only for three months, and the intervention program could cause little residual advantage on social support at six months postpartum. Therefore, suggesting women to retain the ISP access in longer-term may lead to different social support outcomes [33].

The strengths of the research were the sound theoretical framework of ISP, the rigorous research design, and the methodological rigor in data collection and analysis. However, some limitations need to be noted. Firstly, it was not possible to blind researchers during all the research process, which could produce the potential biases. Secondly, the research only approached the participants who were available to the internet, limiting the generalizability of research results. Thirdly, the pilot sample was small which restricted the results. The larger sample RCT study needs to be conducted to further identify the intervention outcomes.

## 5. Conclusions

The research contributed to the practical and scientific knowledge of new parenting intervention method. The effect of ISP was evaluated to significantly increase the levels of MSE, social support, and alleviate PPD symptoms for first-time mothers. However, the small sample in pilot study restricted the research results. Therefore, the ISP should be further investigated with a larger, diverse sample to confirm whether it should be adopted as routine postnatal care to support primiparous women on parenting outcomes and mental wellbeing in the early stage of motherhood. 

## Figures and Tables

**Table 1:** Contents and functions of the ISP.

Components	Functions	Contents
Learning forum	Educational function	⯎ Infant common diseases ⯎ First aid and safety care ⯎ Daily care of infants ⯎ Growth and development ⯎ Breastfeeding and bottle feeding ⯎ Postnatal care for women
Communication forum	Social function	⯎ Parenting feeling, experiences, interactions, etc. ⯎ Belonging and resonance of motherhood ⯎ Topics discussion, such as doing the month and dealing with the relationship with mother-in-law
Ask-the-expert forum	Answering function	⯎ Interaction between experts and mothers ⯎ Questions’ feedbacks ⯎ Baby self-assess tools
Baby home forum	Recording function	⯎ Baby growth and development record ⯎ Baby early educational multimedia resources
Reminder forum	Reminder function	⯎ Learning reminder ⯎ Physical examination reminder ⯎ Questionnaire filling reminder ⯎ Immunization reminder

**Table 2:** Comparison of socio-demographic characteristics and baseline outcomes between the groups.

Variables	Total (*n* = 40)	Intervention Group (*n* = 20)	Control Group (*n* = 20)	t/χ^2^ Value	*p*-Value
Maternal age, mean (SD)	27.25 (3.04)	27.15 (3.15)	27.35 (3.00)	0.013	0.908
Marital status, *n* (%)					
Married	40 (100)	20 (100)	20 (100)	0.000	1.000
Divorced	0 (0)	0 (0)	0 (0)		
Single	0 (0)	0 (0)	0 (0)		
Education, *n* (%)				0.178	0.915
Middle school or lower	9 (22.5)	5 (25)	4 (20)		
High school	15 (37.5)	7 (35)	8 (40)		
University or higher	16 (40.0)	8 (40)	8 (40)		
Occupation, *n* (%)				0.443	0.931
Professional	15 (37.5)	8 (40)	7 (35)		
Skilled	7 (17.5)	3 (15)	4 (20)		
Unskilled	11 (27.5)	6 (30)	5 (25)		
Unemployed	7 (17.5)	3 (15)	4 (20)		
Monthly family income, *n* (%)				0.503	0.778
<3000 yuan (US$420)	5 (12.5)	3 (15)	2 (10)		
3001–5000 yuan (US$420–700)	16 (40.0)	7 (35)	9 (45)		
>5000 yuan (US$700)	19 (47.5)	10 (50)	9 (45)		
Delivery mode, *n* (%)				0.582	0.748
Natural childbirth	22 (55)	12 (60%)	10 (50)		
Assisted childbirth	10 (25)	4 (20%)	6 (30)		
C-section	8 (20)	4 (20%)	4 (20)		
Whether attending parenting train, *n* (%)				0.000	1.000
Yes	18 (45)	9 (45)	9 (45)		
No	22 (55)	11(55)	11(55)		
Baby gender, *n* (%)				0.100	0.752
Boy	21 (52.5)	10 (50)	11 (55)		
Girl	19 (47.5)	10 (50)	9 (45)		
Baby health, mean (SD)	61.42 (18.13)	61.70 (16.76)	61.15 (19.83)	0.701	0.408
Baby fussiness, mean (SD)	49.57 (12.68)	50.20 (12.60)	48.95 (13.05)	1.636	0.209
SICS, mean (SD)	63.61 (7.13)	63.49 (7.17)	63.72 (7.27)	0.023	0.880
EPDS, mean (SD)	5.15 (2.33)	5.05 (2.54)	5.25 (2.15)	0.001	0.982
PSSS, mean (SD)	41.43 (2.78)	41.43 (2.78)	41.43 (2.86)	0.309	0.581

SICS: the Self-efficacy in Infant Care Scale; EPDS: the Edinburgh Postnatal Depression Scale; PSSS: the Postpartum Social Support Scale.

**Table 3:** Effect of the ISP on outcomes at immediately after the intervention (T1), and three months after the intervention (T2).

ISP Effect	Mean (SD) Intervention Group (*n* = 18)	Mean (SD) Control Group (*n* = 18)	Mean Difference (95% CI)	*p*-Value ^a^
Outcomes				
MSE (SICS)				
T1	73.54 (6.38)	66.91 (7.52)	6.63 (1.90 to 11.35)	0.007
T2	72.87 (6.97)	67.12 (7.10)	5.75 (0.98 to 10.51)	0.020
Postpartum depression (EPDS)				
T1	6.11 (2.54)	9.22 (3.30)	−3.11 (−5.11 to −1.12)	0.003
T2	5.78 (2.23)	8.28 (2.66)	−2.50 (−4.17 to −0.83)	0.005
Social support (PSSS)				
T1	45.76 (3.85)	41.45(2.92)	4.30 (1.99 to 6.62)	0.001
T2	42.94 (3.39)	42.59 (3.13)	0.35 (−1.80 to 2.50)	0.743

ISP: Internet-based support program; SICS: Self-efficacy in Infant Care Scale; EPDS: Edinburgh Postnatal Depression Scale; PSSS: Postpartum Social Support Scale; ^a^: All P values were calculated using repeated measures multivariate analysis of covariance

## Data Availability

The data presented in this study are available on request from the corresponding author. The data are not publicly available due to privacy restrictions.
